# Medical and Philosophical Intelligence

**Published:** 1814-12

**Authors:** 


### MEDICAL AND PHILOSOPHICAL INTELLIGENCE.

AT a meeting of the Westminster Medical Society, Mr. EARLE mentioned a case of retention of urine, which had been relieved by an enema of an infusion of Nicotianum. The following were the particulars:—A man had been for some time affected with strictures, but had always been able to void his water in a small stream, until one morning, when he found that none would pass, notwithstanding his bladder was full, and his desire to void urine very urgent. He immediately applied to a neighbouring chemist, who bled him largely, and made several attempts to pass a catheter, and used much force. Not succeeding in his object, he called in Mr. Earle; the patient was in great distress from the distension of the bladder, and bleeding profusely from the urethra. He was ordered to sit in warm water, a clyster of water-gruel was administered, and the Tinctura Ferri Muriate was prescribed in doses of gtt. xv. every ten minutes. This plan was persevered in for four hours without any apparent benefit. Mr. E. now attempted to pass a bougie, but found that it passed out of the urethra when about seven inches down, and took a wrong direction. The bougie was immediately withdrawn, and an injection was directed to be thrown up, consisting of an infusion of tobacco, of the strength of one drachm to eight ounces of boiling water. The effect was very powerful and decided; the patient became faint and sick; his pulse sunk, and his whole body was bathed in perspiration; in a few minutes the urine flowed from him in a small stream. He was directed to keep very quiet, and to bathe the perineum often with warm water. There was no return of the retention, and, after some time had elapsed, bougies were introduced twice a-week, and the strictures much relieved. Mr. Earle further mentioned, that he had resorted to the same remedy in three other cases of obstinate retention, in which no instrument could be passed, in all of which the Enema Nicotianæ had proved eminently useful.

---

At one of the recent meetings of the Medical Society of London, Oil of Turpentine was strongly recommended, as being almost a panacea for *acute* Rheumatism. The formula in which it was administered with so much success is—Ol. Terebinth. gtt. xx. Decoct. Cinchon. ℥ifs. 4tis. horis. The use of the lancet and purgatives, we believe, were generally premised. No sensible operation ensued from the medicine; but the patients were quickly relieved of the complaint.

---

A case has been communicated to the Medico-Chirurgical Society, of the mechanical cure of that species of incontinence of urine occasioned by sloughing of the lower surface of the bladder and vagina, consequent to parturition. The contrivance employed consists in a tube of elastic gum passed into the vagina, upon which which is fixed a piece of sponge corresponding in size and situation with the opening into the bladder; the sponge is then introduced into the aperture, which, by the pressure of the elastic tube, is closed so as to allow no passage for the flow of urine through it. By means of a female catheter, the water is very frequently drawn off, or the bladder kept constantly emptied; the urine thus diverted into its natural channel, no longer prevents the healing of the ulceration, which gradually takes place. As the sponge absorbs part of the urine, the surface which is applied to the ulcerated margins of the aperture should be smeared with ointment, or some substance capable of keeping the urine from them. Where the incontinence arises from paralysis of the sphincter urethræ, of course this practice is not likely to be beneficial.

---

The Committee of Medical and Physical Science of the Society of Emulation and Encouragement of the Arts and Sciences at Liege, have offered a Gold Medal to the author of the best Dissertation on indigenous Vegetable Poisons. Four questions are submitted to the notice of the candidates: 1. What are the principles in which the deleterious properties reside? 2. What is the mode of action which they exercise on the animal economy? 3. What are the organic lesions which they produce? 4. What are the best means of obviating their bad effects?

The same Committee offers a Silver Medal to the author of the best Medical Topography of a canton in the department of the Ourthe. Memoirs to be addressed, free of postage, to M. DE JAER, Liege.

---

Mr. BECLARD has made some observations in the Bulletin de la Faculté de Medicine, which seem to prove that the fœtus breathes the liquor amnii. When he first entered upon the investigation of this question, he was not aware that it had occupied the attention of any inquirers since the time of Haller; and was first led to the consideration of it by the opinion of Rœderer, and by that promulgated by Professor Dubois, in his lectures on midwifery. He has since become acquainted with the inquiries prosecuted at Copenhagen by Winslow, Scheele, Riegel, Heroldt, Abildgard, and Wyburg, but his conclusions differ in some respects from theirs.

If we examine a still-born fœtus, we may easily convince ourselves by dissection, that the trachea and the bronchi are filled with a fluid resembling the liquor amnii. Fœtuses which are born feeble, and with the face upwards, evacuate a large quantity of liquid during the first moments in which they breathe the air; they are frequently obliged to be placed upon the side, in order to favour this evacuation; and sometimes it is necessary to clear the mouth and the pharynx. When the infant is strong, and is born with the face upwards, it frees itself from this liquid in a few instants. Mr. B. once had occasion to perform the Cæsarian operation upon a woman who had just expired; the fœtus was also dead, and had the mouth, the pharynx, the trachea, and the bronchi, bronchi, filled with water. He has several times opened the pregnant female of different animals; after having cut into the uterus, he tied the neck of several fœtuses, whilst still enclosed in the amnios, and on examining the trachea and bronchi, it was found then filled with a liquid similar to the liquor amnii. To clear up any doubts that might remain upon the subject, water coloured black by the addition of ink was injected into a small opening in the amnios of a bitch; after a few moments, he tied the neck of the fœtus, which was still living, and he found the black fluid in the trachea and the bronchi.

In cases of presentation of the feet, every practitioner in midwifery has undoubtedly seen and felt the well-marked motions of inspiration which are made by a vigorous fœtus, when the head, the neck, and the superior members, are still contained in the uterus and vagina.

When the uterus of the pregnant female of any animal is laid open with caution, the motions of respiration, by which he means the opening of the mouth, and dilatation of the nostrils, synchronous with the elevation of the parieties of the thorax, may be distinctly observed through the membranes and the liquor amnii: they are repeated at regular intervals; they are in general slower than the motions of respiration in animals of the same species breathing the atmosphere; they become more frequent as the circulation between the mother and the fœtus becomes imperfect by the gradual contraction of the uterus; they bear a striking resemblance in general to the rare and deep respiratory motions of fœtuses which are born in a state of weakness, and approaching to suffocation. It is yet uncertain whether, besides the mechanical phenomena of respiration, which evidently exist in the fœtus, particularly when any obstacle is opposed to the circulation through the funis, any chemical action takes place between the liquor amnii and the blood, which passes through the lungs, in quantities proportionally greater as the period of birth approaches.

With respect to the introduction of the liquid into the intestinal canal, Mr. B. possesses some facts in addition to those by which Haller has established this opinion. He examined some meconium with Professor Chaussier, and found in it hairs similar to those which are met with upon the skin of the fœtus, in the latter part of its existence in the uterus. He dissected the body of a fœtus in which the intestinal canal was in one part obliterated; the superior part only contained meconium, the inferior contained only a sweetish colourless mucus.

---

The Gazette de France gives some curious particulars of experiments made with a new diving machine by Mr. Melville the inventor. He descended twice in the Seine, near the Pont Royal, to the depth of from 10 to 20 feet, and passed 56 minutes at the bottom. He took with him two swans, two ducks, and some bread and wine. He let loose the aquatic animals while under water, went from the Pont Neuf to the Swimmer's Aquatic School, and came out dressed as usual, without being in the least wet. The machine does not resemble any thing of the kind hitherto employed. It is neither a barrel or a bell, but has rather the form of an egg. It is not bulky, contains only five cubic inches of air, so prepared that pressure can do it no harm; but it is kept pure and fresh. Mr. M. has taken with him cats, rabbits, dogs; but the latter cannot bear this kind of air longer than five minutes, as they go mad in it; but he declares he could stay half a day in his machine under water, without the slightest inconvenience. He has the use of all his limbs, and can do what he pleases—saw wood, bore gimblet holes, and pick up the smallest objects. His pulse rises from 120 to 160, and he feels from it an agreeable sensation, a kind of electrical effect.

---

On the establishment of peace between France and this country, a Frenchman, by name Pradier, advertized a certain cure of gout by his newly-discovered medicine. Curiosity led us to inquire into the particulars of his secret. It consisted of a poultice of linseed meal, sprinkled with a spirituous liquid, and made sufficiently large to cover the parts affected. It appears, from the French journals, this remedy had then been purchased by their government, and the following farrago is the certified prescription.

Balsam of Mecca, six drachms; red bark, an ounce; saffron, half an ounce; sago, an ounce; sarsaparilla, an ounce; alcohol, three pounds: mix for a tincture. About ℥ ij. of this liquid is diffused over the surface of a *hot* poultice, of a large size, and applied to the parts. It may be changed every twelve hours.

The use of cataplasms in arthritic complaints is not a new practice. Riolan (the father) recommends from Galen one made of fœnugreek powder, honey, and vinegar, which in three days produces wonderful effects "fiat hoc triduo & miraberis effectus." Emollient applications have always had their advocates; but, as it has been hinted by some, and especially by Baglivi and Barthez, that they leave the parts in a state of greater laxity, they have not been very universally employed. The latter of these authors, in his Treatise on Gout, has collected a great number of analagous formulæ, and advises the choice of those which assist the transpiration of the part.

The Commissioners of Secret Remedies in France have had several specific poultices presented to them for the cure of gout. One of them is made with the meal of pease, which very nearly approximates to our *pease-pudding*. Another, for the soles of the feet, is made with turnips, rice, and rye flour, impregnated with different saline and mineral substances. One individual proposes nettles boiled in wine. It is not uncommon on the continent to use large and thick cataplasms, with tincture of saffron or guaicum sprinkled on them.

If there be any virtue in Mr. Pradier's remedy, we suspect the whole is to be ascribed to the alcoholic spirit and poultice, which

3 x 2 are are not unlikely to be serviceable in some cases. The sum of 6000 francs was agreed to be given to him for its publication.

---

In a former Number we noticed the experiments of M. BERTRAND, which tended to prove that charcoal was an antidote to certain mineral poisons. These, however, have been repeated by M. ORFILA, who authorizes the editor of the Gazette de France to announce his intention of refuting them in his ensuing publication on the subject of Poison.

---

*Cancerous Ulcers of the Nose cured by Rousselot's Powder.*—A woman, 66 years of age, had for seven years a carcinomatous ulcer, from the basis of which arose fungous excrescences of a horny nature. M. PETIT covered the surface with the powder, (consisting of oxyde of arsenic, 1 part; dragon's blood, 8 parts; and 16 parts of sulphuret of mercury.) The eschar produced by it fell off at the end of three days, and exposed a red and inflamed surface. After suppuration was established, the inflammation abated, and the powder was applied a second time. The eschar now formed fell off in four days, when the disease required nothing but simple cerate for its complete cure. Another case of the same kind was cured by three applications.

---

The boy we mentioned in one of our former Journals as the subject of Elephantiasis in St. Bartholomew's, now labours under measles, a further proof of the justice of Dr. Christie's remark, that this dreadful disease, in its worst form, is no protection against contagion.

---

Mr. SANKEY has published some Observations on Phlegmatia Dolens. He has never seen it follow the first labour, or attack the same patient twice; two cases were in young women, not after parturition: both were severe, and well marked; both had obstructed menses, and one had a suppression of urine. One well-marked case existed in a man 60 years old. In Dr. Wyer's, and in most of the cases described by Dr. Hull, Dr. Ferriar, and Dr. White, it was confined to one leg only. Most of the patients seen by Mr. S. have had both legs affected, not at the same time, but, after going through the progress in one, the other becomes affected; and (unless prevented by the application of blisters) goes through the same stages, and takes the same time as the first. He has always found gentle laxatives useful and necessary, even when there is diarrhœa. To ease the pain, small doses of opium with antimony have been given; every other internal treatment has been directed to palliate symptoms. As there is always considerable debility, accompanying or following—generally want of appetite, attended with a quick and feeble pulse,—he gives some kind of tonic as soon as the stomach and state of fever would admit; but all these seem only auxiliaries, and not very important, except opening the bowels, and moderate doses of opium. What he he considers to be specific, is a blister applied to the calf of the leg immediately upon discovering the complaint. When the symptoms have made some progress, it is often some time before the complaint can be subdued, though every blister is seen to relieve the pain, and, when thus completely formed, it often requires several blisters. The first is applied to the calf of the leg, as the pain is generally most severe in that part, and there is less fear of its not healing than if applied lower. If required, they are repeated every two or three days, not at the same place, but higher or lower, according to the seat of the pain. With every attention, this complaint often leaves considerable weakness in the leg most affected, requiring bandage or laced stocking. Though it is often a tedious and painful complaint, he has never seen it fatal.

---

Mr. HARTTE has related a case of Hemiplegia, treated with Tincture Lyttæ, and cold Shower-bath. —— Hope, a private soldier in his Majesty's 1st West India Regiment, (while at Trinadad,) was admitted into the hospital on the 29th June 1812, with Cachexia Africana, for which bark, steel, and other tonic medicines were given. He appeared to be recovering; but, on the morning of the 18th of July, it was found, that, from paralysis, he had lost the power of his right side; that he appeared to have no sense of feeling in it, or the extremities of that side; his articulation was scarcely to be understood; he seemingly did not suffer pain, yet he would frequently with his left hand take hold of his tongue, as if with a view of squeezing it smaller. Blisters were applied between the shoulders, along the vertebræ of the neck, as well as the region of the sacrum; asafœtida and other antispasmodics were employed with success. On the 29th July, five drops of the tinct. lyttæ were given three times a-day, increasing the dose one drop daily.

On the 3d August, ten drops produced ardor urinæ: the medicine was discontinued for one day, and rice-water ordered for common drink, and the region of the bladder was fomented. The following day, (5th August,) he commenced the tincture with ten drops (ter in die). On the 16th August, he was sensible when any part of his side or extremities was touched, and his articulation began to be more distinct; on the 20th August he could stand with the assistance of a stick. On the 1st September, the cold shower-bath was ordered every morning. When at 37 drops per day, he could make some steps without the stick: this quantity was given until the 20th September, when he was so convalescent, that it was discontinued, and the cold shower-bath only was used. On the 4th October he was discharged in perfect health.—*Edin. Journ.*

---

*Protrusion of the Brain.*—Dr. Sibley, of Union, has communicated a case of fractured cranium, in which the dura mater was wounded and a small portion of the substance of the brain lost. The patient was trepanned by Dr. Brown of Waldoborough; he was delirious delirious a short time after the operation, and on the fourth day the brain began to protrude from the wound and continued to increase to the height of an inch, at the same time suppurating freely. Being lessened to half its size by the suppuration, pressure was applied, which in a few days reduced it wholly, and the boy soon recovered his health of body and mind.—*New England Journal.*

*Spina Bifida.*—Dr. Sewall of Ipswich informs us of a case of spina bifida, which, at the age of two years was accompanied with a tumour at the fontenelle, which by pressure could be made to disappear, while the tumour on the spine would be proportionately enlarged. In consequence of the solicitation of the parents, this tumour was punctured and on its being emptied, that at the fontenelle also subsided. Convulsive respiration immediately succeeded the operation, in six hours spasms, and at the end of twelve the child expired.—*New England Journal.*

*Foreign Substance in the Lungs.*—Dr. Buck, of Wilmington, has given an account of a case, in which a boy about two years old was suddenly seized with symptoms of a severe pulmonic affection, which continued occasionally by severe, suffocating paroxysms to reduce his strength for above three months; when he was so exhausted as to be continually threatened with death. A violent fit of coughing and suffocation induced his mother to give him, according to common practice, a smart blow on the back, which had the effect of driving a water melon seed from the trachea; the patient was relieved and recovered.—*New England Journal.*

Dr. Davis, Physician to the Queen's Lying-in Hospital, will commence his second winter Course of Lectures on the Theory and Practice of Midwifery, and on the Diseases of Women and Children, on Wednesday, January the 4th, 1815.

Dr. Adams is preparing an Illustration of Mr. Hunter's Doctrine concerning the Vitality of the Blood, in answer to the Edinburgh Review of Mr. Abernethy's Lectures.

A Statement of the early Symptoms which lead to the Disease termed Water in the Brain, with Observations on the necessity of a watchful Attention to them, and on the fatal consequences of their Neglect; in a Letter to Martin Wall, M.D. by G. D. Yeats, M.D. of Trinity College, Oxford, of the Royal College of Physicians, London, late Physician to the Infirmary and Lunatic Asylum of the County, and Physician to his Grace the Duke of Bedford; will shortly be published.

Remarks on the Symptoms and Treatment of the Diseased Spine previous to the period of Incurvation, illustated with two Engravings, by Thomas Copeland, M.D. Fellow of the Royal College of Surgeons, and Assistant-surgeon to the Westminster General Dispensary, is also in the press.

METEO-

